# Alzheimer’s disease: a continuum with visual involvements

**DOI:** 10.3389/fpsyg.2023.1124830

**Published:** 2023-07-06

**Authors:** Lorena Elvira-Hurtado, Inés López-Cuenca, Rosa de Hoz, Mario Salas, Lidia Sánchez-Puebla, Federico Ramírez-Toraño, José A. Matamoros, José A. Fernández-Albarral, Pilar Rojas, Soraya Alfonsín, María Luisa Delgado-Losada, Ana I. Ramírez, Juan J. Salazar, Fernando Maestu, Pedro Gil, José M. Ramírez, Elena Salobrar-García

**Affiliations:** ^1^Ramon Castroviejo Institute for Ophthalmic Research, Complutense University of Madrid, Madrid, Spain; ^2^Health Research Institute of the Hospital Clínico San Carlos (IdISSC), Madrid, Spain; ^3^Faculty of Optics and Optometry, Department of Immunology, Ophthalmology and ENT, University of Madrid, Madrid, Spain; ^4^Memory Unit, Geriatrics Service, Hospital Clínico San Carlos, Madrid, Spain; ^5^Center for Cognitive and Computational Neuroscience, Laboratory of Cognitive and Computational Neuroscience, Complutense University of Madrid, Pozuelo de Alarcón, Spain; ^6^Department of Experimental Psychology, Cognitive Psychology and Speech and Language Therapy, Complutense University of Madrid, Pozuelo de Alarcón, Spain; ^7^Madrid Eye Institute, Gregorio Marañón General University Hospital, Madrid, Spain; ^8^Department of Medicine, School of Medicine, Complutense University of Madrid, Madrid, Spain; ^9^Faculty of Medicine, Department of Immunology, Ophthalmology and ENT, University of Madrid, Madrid, Spain

**Keywords:** Alzheimer’s disease, mild cognitive impairment, family history, visual function, visual acuity, contrast sensitivity, PDT

## Abstract

**Introduction:**

Alzheimer’s disease (AD) is the most common form of dementia affecting the central nervous system, and alteration of several visual structures has been reported. Structural retinal changes are usually accompanied by changes in visual function in this disease. The aim of this study was to analyse the differences in visual function at different stages of the pathology (family history group (FH+), mild cognitive impairment (MCI), mild AD and moderate AD) in comparison with a control group of subjects with no cognitive decline and no family history of AD.

**Methods:**

We included 53 controls, 13 subjects with FH+, 23 patients with MCI, 25 patients with mild AD and, 21 patients with moderate AD. All were ophthalmologically healthy. Visual acuity (VA), contrast sensitivity (CS), colour perception, visual integration, and fundus examination were performed.

**Results:**

The analysis showed a statistically significant decrease in VA, CS and visual integration score between the MCI, mild AD and moderate AD groups compared to the control group. In the CS higher frequencies and in the colour perception test (total errors number), statistically significant differences were also observed in the MCI, mild AD and moderate AD groups with respect to the FH+ group and also between the control and AD groups. The FH+ group showed no statistically significant difference in visual functions compared to the control group. All the test correlated with the Mini Mental State Examination score and showed good predictive value when memory decline was present, with better values when AD was at a more advanced stage.

**Conclusion:**

Alterations in visual function appear in subjects with MCI and evolve when AD is established, being stable in the initial stages of the disease (mild AD and moderate AD). Therefore, visual psychophysical tests are a useful, simple and complementary tool to neuropsychological tests to facilitate diagnosis in the preclinical and early stages of AD.

## 1. Introduction

Alzheimer’s disease (AD) is a neurodegenerative disease that affects the central nervous system and is the most common cause of dementia in the world (Cunha et al., 2016). This neurodegenerative disease is histologically characterized by the accumulation of beta-amyloid (Aβ) and intracellular neurofibrillary tangles of hyperphosphorylated tau protein (pTau) (Walker, 2020). The main risk factor for developing AD is the age (Capizzano et al., 2004; Chen et al., 2009), following this, two of the genetic risk factors are: having a first-degree family history of AD and carrying at least one ε4 allele for the ApoE gene (Sano et al., 1991; Donix et al., 2010). These subjects with a parent with AD have a 4 to 10 times higher risk of developing the disease (Huang et al., 2004). Some of these high-risk individuals may develop the pathology over time. Therefore, it could be considered that these subjects may be in a preclinical phase, since the first biochemical changes occur even 20 years before cognitive decline appears.

AD is a continuum because it is a progressive neurological disorder that develops gradually over time. This continuum begins when the subject is cognitively healthy, although there are molecular changes that already foreshadow the progression toward cognitive decline, to a phase in which the patient has cognitive impairment that cannot be independent in their daily life (Alzheimer’s association, 2020; Alzheimer’s Association, 2022). MCI can be considered as the stage of cognitive status prior to AD (Sperling et al., 2011). MCI is characterized by impairment of cognitive functions in the performance of everyday activities, that is greater than what would be expected for a person’s age and education level, but not severe enough to interfere significantly with daily activities or independent functioning (Sanford, 2017; Alzheimer’s Association, 2022). When dementia is established current criteria describe three stages: mild AD, moderate AD and severe AD (McKhann et al., 2011; Alzheimer’s Association, 2022).

The retina is a projection of the brain, and it is known that in some brain neurodegenerative diseases there are retinal changes (Ramirez et al., 2017; Colligris et al., 2018; Salobrar-García et al., 2019; Rojas et al., 2020a,b; Alves et al., 2023). In recent decades, numerous studies have investigated retinal alterations in AD, observing that there is neuronal death that first affects the macular region in early stages of the disease, and later, as AD progresses, it affects the peripapillary region of the retina (Blanks et al., 1989; Garcia-Martin et al., 2014; Salobrar-García et al., 2015, 2019; Koronyo et al., 2017). These structural changes are often accompanied by functional visual changes, which have been described in patients with MCI and AD compared to healthy subjects, reporting decreased visual acuity (VA) and contrast sensitivity (CS), as well as poorer colour perception and impaired visuospatial integration (Sadun et al., 1987; Risacher et al., 2013, 2020; Salobrar-García et al., 2015, 2019; Chang et al., 2022). These visual functional tests are easy to perform, quick and non-invasive and can provide information on the onset, stage and evolution of the neurodegeneration.

These changes in visual function can have a significant impact on the daily lives of individuals with AD and their caregivers. It is important to monitor and manage visual changes in individuals with AD to help maintain their quality of life.

Currently, the continuum of AD is well understood in terms of biochemical changes, brain atrophy, and cognitive decline. However, what is not clearly established is how visual function changes in these patients throughout the evolution of the pathology, from preclinical stages to advanced disease. It is therefore crucial to understand the visual changes that occur in these patients, given their importance for maintaining independence in daily life. By gaining a better understanding of visual changes in the context of the disease continuum, we can improve patient care and outcomes. Despite the existence of numerous reports detailing the examination of retinal biomarkers in AD, as evidenced by numerous articles and reviews, the literature concerning the evaluation of visual perception in AD remains limited. This may be attributed to the challenge of obtaining subjective measurements in individuals with cognitive decline. Nonetheless, investigating these visual perceptual variables in early-stage cases may be worthwhile in order to determine whether they can be utilized as supplementary biomarkers for AD. Thus, the aim of this study is to analyze the differences in visual function in the different stages of the disease continuum, from healthy subjects with high genetic risk for the development of the disease to patients with moderate AD.

## 2. Materials and methods

### 2.1. Subjects

The study subjects were recruited from the Memory Unit of the Hospital Clínico San Carlos in Madrid and from the COGDEM study “The cognitive and neurophysiological characteristics of subjects at high risk of developing dementia: a multidimensional approach.”

All patients were ophthalmologically examined at the Ramon Castroviejo Institute for Ophthalmic Research clinic of the Complutense University of Madrid and signed the informed consent form. The research followed the tenets of the Declaration of Helsinki, and the studies were approved by the local ethics committee (HCSC) with the internal code 11/372-E, 18/422-E_BS, and 20/698-E_Tesis.

**Figure 1:**
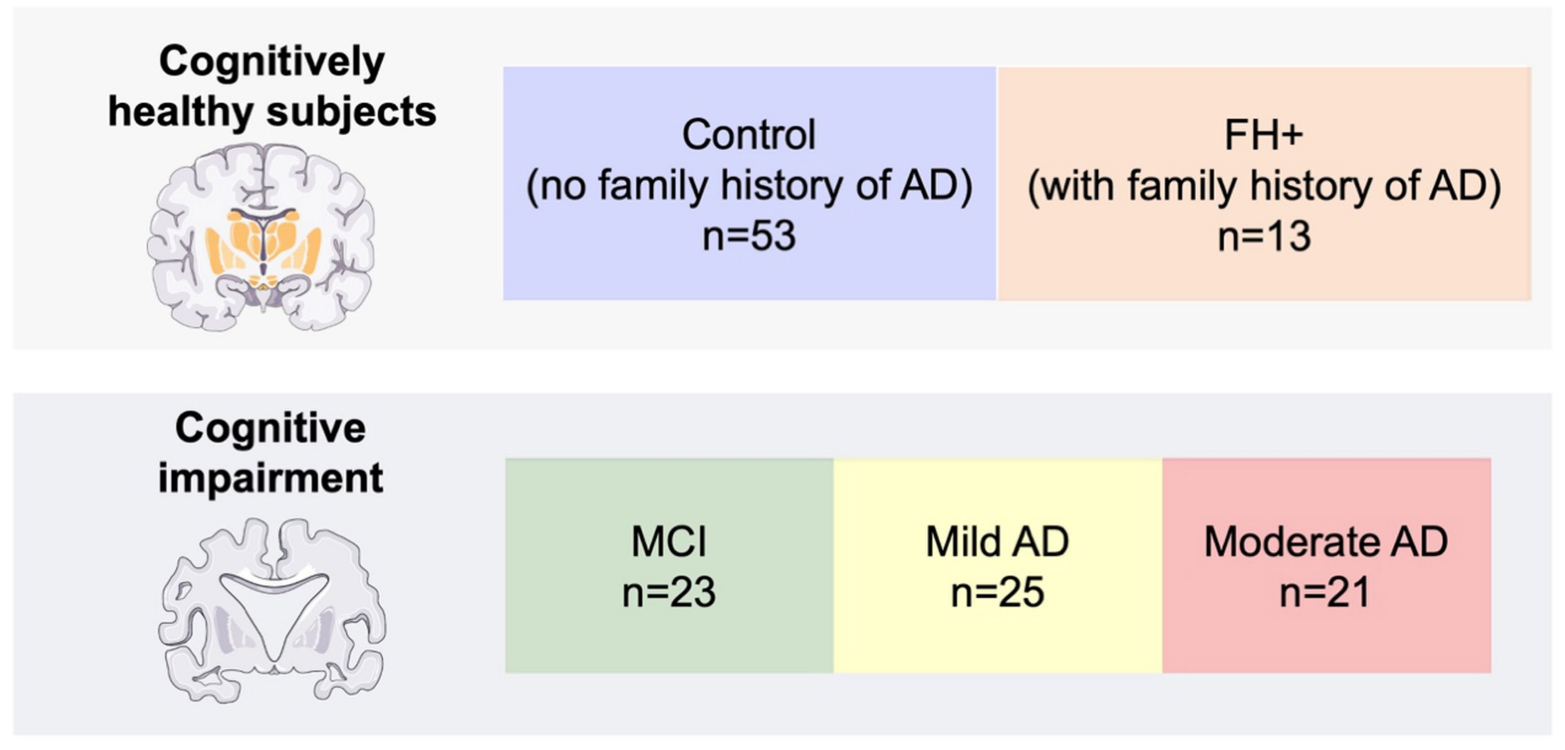
Number of participants per group. FH+, family history positive; MCI: mild cognitive impairment; AD, Alzheimer’s disease.

The cognitively healthy subjects were divided into two groups: (i) controls with no family history of AD (*n* = 53); (ii) healthy subjects with a first-degree history of AD (*n* = 13) (Figure 1). Cognitively healthy subjects had a normal T2-weighted brain magnetic resonance imaging (MRI) without evidence of brain injury or pathology and a Mini Mental State Examination (MMSE) above 26.

Subjects with cognitive impairment were categorized according the National Institute of Neurological and Communicative Disorders and Stroke-AD and Related Disorders Association and the Diagnostic (NINCDS-ADRDA) and Statistical Manual of Mental Disorders V (DSM V) to the guidelines into patients with MCI (*n* = 23), mild AD (*n* = 25), and moderate AD (*n* = 21) (Figure 1). All cognitively impaired participants had a MMSE score between 25 and 17.

All participants had no history of neurological or psychiatric disorders or a serious medical condition and being free of systemic disorders affecting vision in their medical record. In all study groups, subjects met the following ophthalmologic inclusion criteria: be free of ocular disease or posterior pole pathology (macular degeneration, drusen, glaucoma or suspected, epiretinal membrane, congenital malformation) have best corrected VA better than 0.5 decimal, have less than ±5 spherocylindrical refractive error, have intraocular pressure less than 20 mmHg.

### 2.2. Ophthalmological tests

The complete ophthalmologic examination included: measurement of VA, refraction, slit lamp examination, applanation tonometry (Perkins MKII tonometer, Clement Clarke International, Essex, England), CS analysis with CSV-1000E, colour perception test with Farnsworth 28 Hue test, perception digital test (PDT), fundus examination and optical coherence tomography (OCT).

The analysis of visual function was performed through the results obtained in psychophysical tests which are described below.

#### 2.2.1. Visual acuity

The best corrected VA was determined using the Snellen test (decimal scale), as previously described by Salobrar-García et al. (2015). The subjects have to identified the set of letters of each VA level up to his or her maximum, recognizing at least five letters out of eight in a given row, that is the point of highest gradient on the psychometric acuity function approximately of 56.25%. Decimal VA is expressed as a decimal number, where 1.0 represents normal vision, and lower numbers indicate poorer vision.

#### 2.2.2. Contrast sensitivity

To analyze CS, it was used CSV-1000E system (VectorVision, Greenville, OH, United States) with the patient’s best corrected VA. The manufacturer’s recommendation for viewing distance and illumination levels was followed and four spatial frequencies [3, 6, 12, and 18 cycles per degree (cpd)] were analysed. The result provides us with a sensitivity curve in logarithmic values that is provided by the manufacturer.

#### 2.2.3. Colour perception test

It was used the Farnsworth 28-Hue test (Luneau, Paris), that is a color vision test that measures a person’s ability to distinguish differences in color hues. It consists of 28 color tiles, arranged in four rows of seven tiles each, that must be arranged in a specific color order. The test is used to assess color vision deficiencies, such as color blindness, and to evaluate a person’s color discrimination ability. During the test, there were no time restrictions and the subject was permitted to make corrections. To determine the extent of tritan and deutan errors, the manufacturer’s manual blue axis errors were taken into consideration when caps 43 to 64 were malpositioned, and deutan axis errors were considered for caps 42 to 85.

#### 2.2.4. Perception digital test

Perception digital test (PDT) (Rami et al., 2007) was used to analyse visual integration. It is a quick, simple and sensitive test used in patients with AD. The test consists of 15 slides. Each slide shows the same distorted image (special effects: geometric effects (tile) and the effect of the frame 24/48 of the MGI Photo Suite III program) in different positions in space. The patient had to identify the image that was correctly oriented in space.

### 2.3. Statistical analysis

Statistical analysis was performed in Prism 9.0.1 (GraphPad Prism, La Jolla, CA, United States). Data were indicated as median ± interquartile range. The Kruskal-Wallis test with the Dunn’s test for multiple comparisons was used to analyze differences between the study groups (control, FH+, MCI, mild AD and moderate AD) in VA, Colour vision and PDT; CS was analyze using 2way-ANOVA test with the Tukey multiple comparison test. The sensitivity at 90% specificity and the area under the receiver operator characteristic (aROC) analysis were computed for all the psychophysical tests that were examined, with the aim of distinguishing between healthy individuals and those diagnosed with AD. Furthermore, these calculations were performed to compare the control group with the other groups. Correlation was applied using Spearman’s correlation coefficient to study the possible association between MMSE and visual function test (VA, CS, color vision and PDT). A *p*-value of <0.05 was considered statistically significant after correction for multiple comparison. The notations used for the different levels of significance were *p < 0.05, **p < 0.01, ***p < 0.001.

## 3. Results

### 3.1. Demographic analysis

When we analyzed age between the study groups, we found statistically significant differences (value of *p*<0.01) between the control group and: (i) FH+ group; (ii) MCI group and; (iii) moderate AD group. We also found significant differences (value of *p*<0.01) between FH+ group and: (i) MCI group; (ii) mild AD group and; (iii) moderate AD group (Table 1).

**Table 1:** Demographics variables of the study.

Groups demographic variables	Control (*n* = 53)	FH+ (*n* = 13)	MCI (*n* = 23)	Mild AD (*n* = 25)	Moderate AD (*n* = 21)
Age	75.00 (72.00–78.00)	68.00 (66.00–70.50)	79.00 (75.75–83.25)	76.00 (75.00–79.00)	77.00 (75.00–81.00)
Sex (*n* = Male/Female)	(*n* = 22/31)	(*n* = 5/8)	(*n* = 11/12)	(*n* = 10/15)	(*n* = 6/15)
MMSE	29.00 (28.00–30.00)	29.00 (29.00–30.00)	29.00 (26.00–30.00)	25.00 (21.00–26.50)	19.00 (18.00–23.00)

Median (interquartile range) MMSE, mini mental state examination; FH, family history positive; MCI, mild cognitive impairment; AD, Alzheimer’s disease.

**Figure 2:**
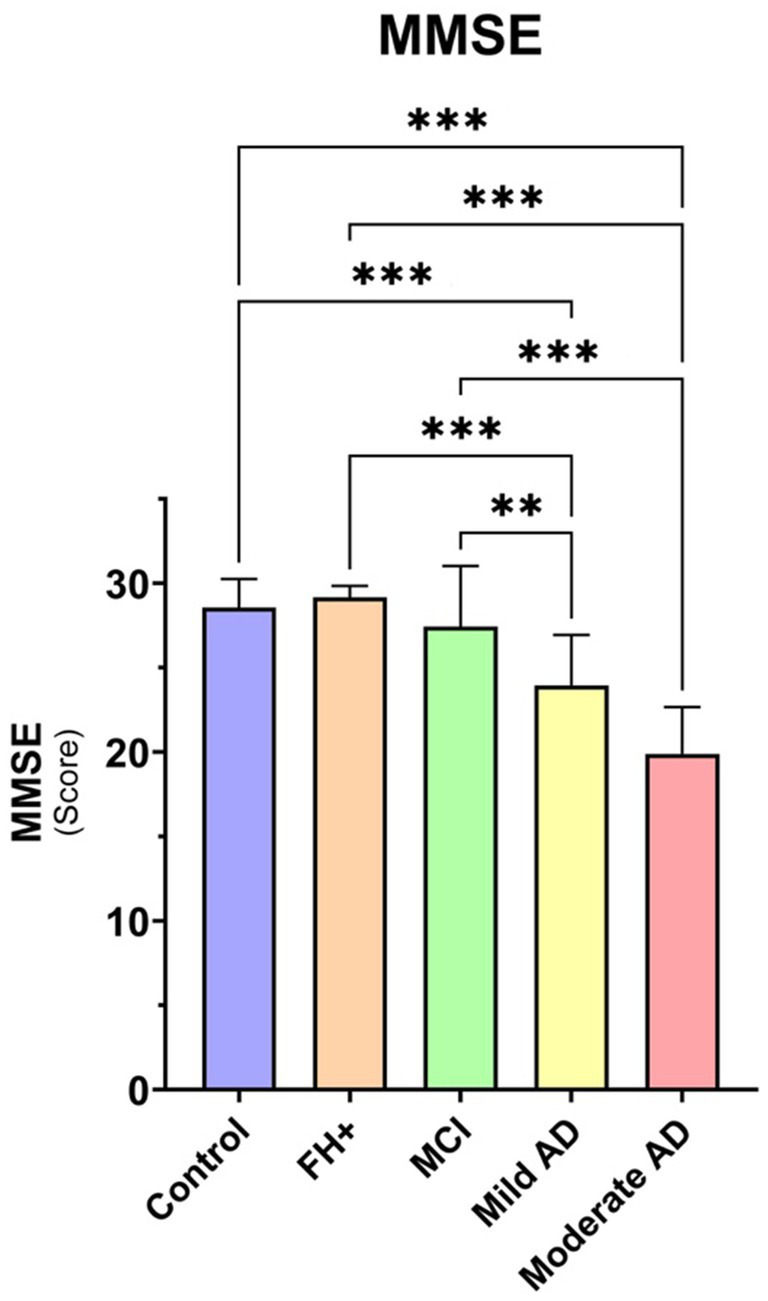
Median data of MMSE score in the study groups. FH+, family history positive; MCI, mild cognitive impairment; AD, Alzheimer’s disease. Each bar represents the media*n* ± interquartile range. **p < 0.01; ***p < 0.001.

The MMSE scores showed significant differences (value of *p*<0.001) between the control, FH+ and MCI groups compared with: (i) mild AD and; (ii) moderate AD (Table 1; Figure 2).

### 3.2. Visual acuity

**Figure 3:**
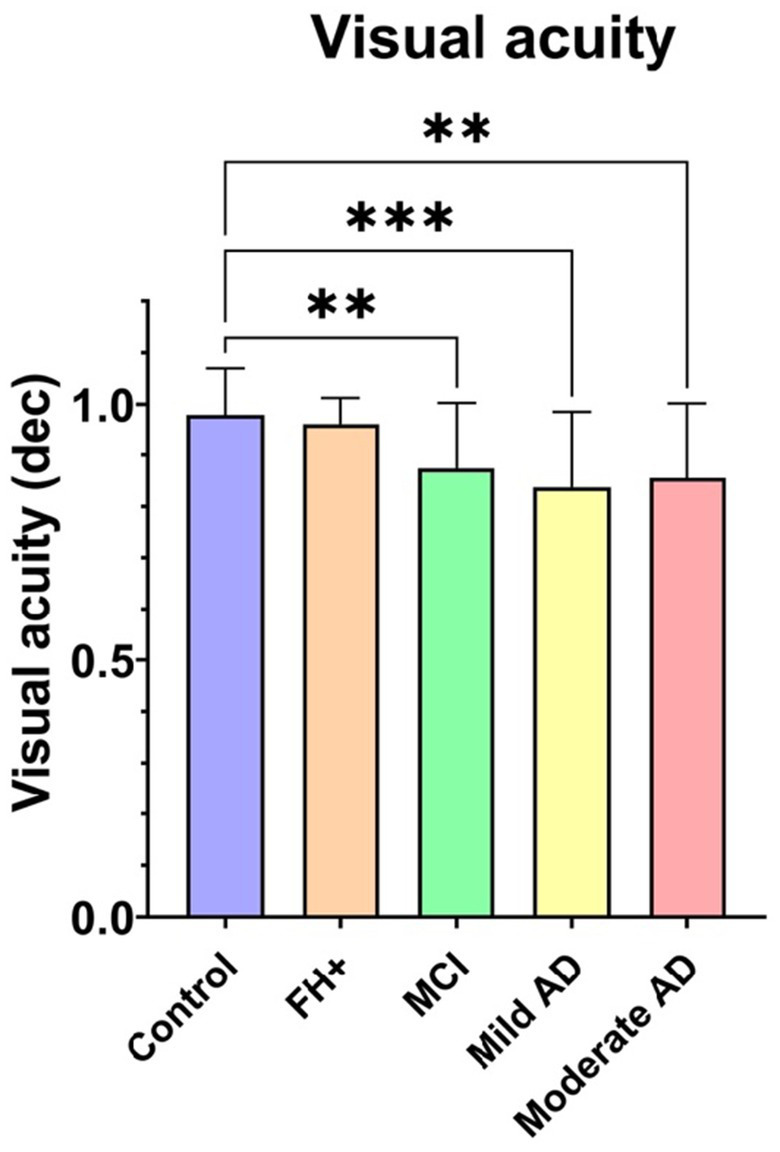
Median data of visual acuity in the study groups. FH+, family history positive; MCI, mild cognitive impairment; AD, Alzheimer’s disease; dec, decimal. Each bar represents the media*n* ± interquartile range. **p < 0.01; ***p < 0.001.

The VA analysis showed a statistically significant decrease (*p* < 0.001) between the MCI, mild AD and moderate AD groups with respect to the control group (Figure 3).

### 3.3. Contrast sensitivity

**Figure 4:**
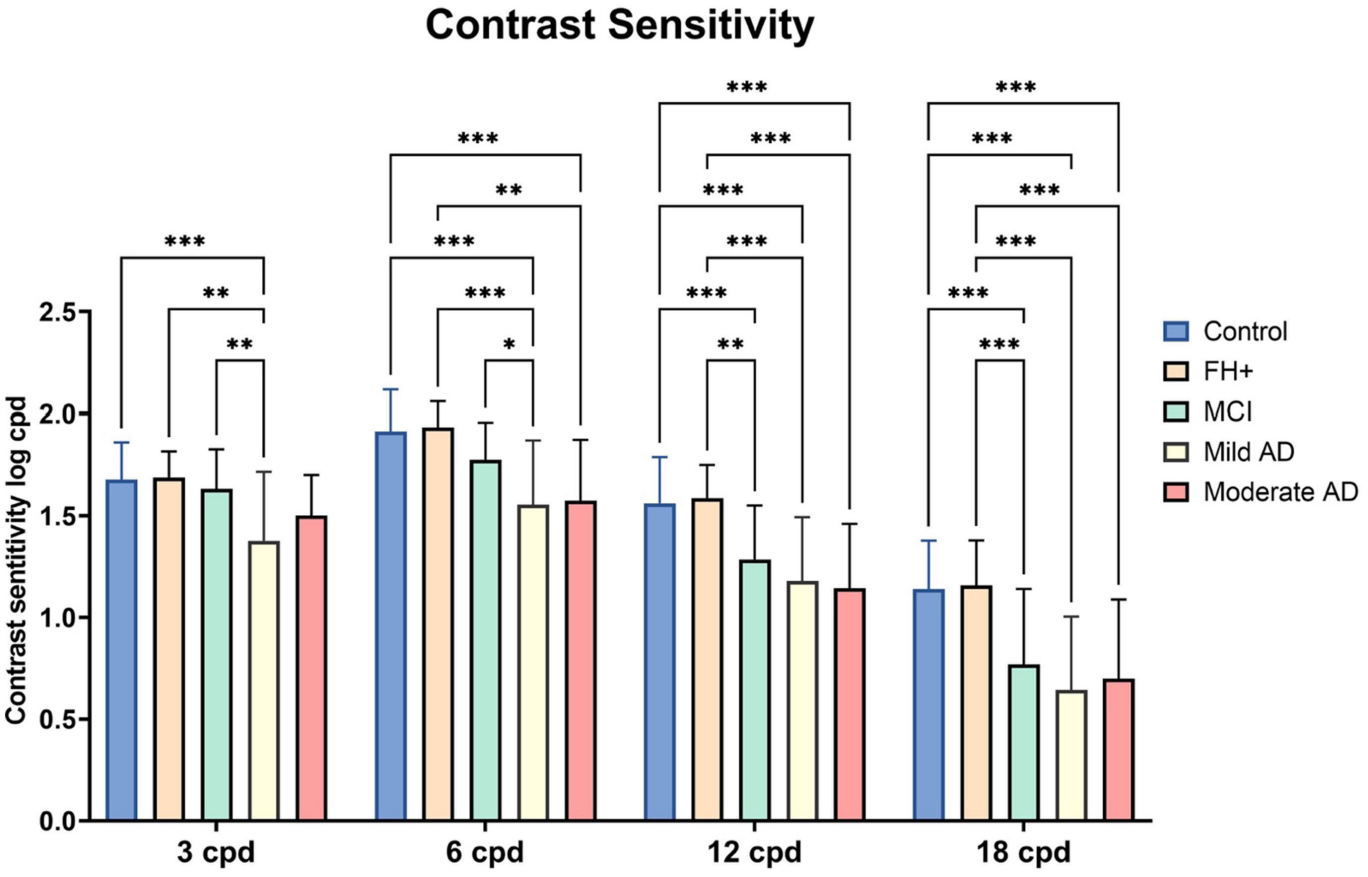
Median values of contrast sensitivity in the different groups. FH+, Family history positive; MCI, mild cognitive impairment; AD, Alzheimer’s disease; CS, contrast sensitivity; cpd, cycles per degree. Each bar represents the media*n* ± interquartile range. *p < 0.05; **p < 0.01; ***p < 0.001.

When comparing CS at the spatial frequency of 3 cpd, we found statistically significant differences between mild AD group and: (i) control group (value of *p*<0.001); (ii) FH+ group (value of *p*<0.01); and; (iii) MCI group (value of *p*<0.01) (Figure 4).

We observed a statistically significant differences in the 6 cpd contrast sensitivity (CS) between the control group and both the mild AD group and moderate AD group (value of *p*<0.001, in both) (Figure 4). Additionally, we observed a significant decrease in the mild AD group (value of *p* < 0.001) and moderate AD group (value of *p* < 0.01) in comparison with the FH+ group (Figure 4). Also, there is a decrease in the VA in the mild AD group compared with the MCI group (value of *p* <0.05) (Figure 4).

At the spatial frequency of 12 cpd, we observed statistically significant differences between the control group and: (i) MCI group, (ii) mild AD group and; (iii) moderate AD group (*p* < 0.001, in all instances). In addition, we also found a significant decrease when comparing the FH+ group and: (i) MCI group (*p* < 0.01); (ii) mild AD group (*p* < 0.001) and; (iii) moderate AD group (*p* < 0.001) (Figure 4).

Finally, significant differences were observed at a spatial frequency of 18 cpd between the control group and: (i) the MCI group, (ii) mild AD group, and (iii) moderate AD group. Additionally, compared to FH+ group a significant decrease (*p* < 0.01) was found in: (i) the MCI group, (ii) mild AD group, and (iii) moderate AD group (value of *p*<0.001, in all instances) (Figure 4).

### 3.4. Color perception

**Figure 5:**
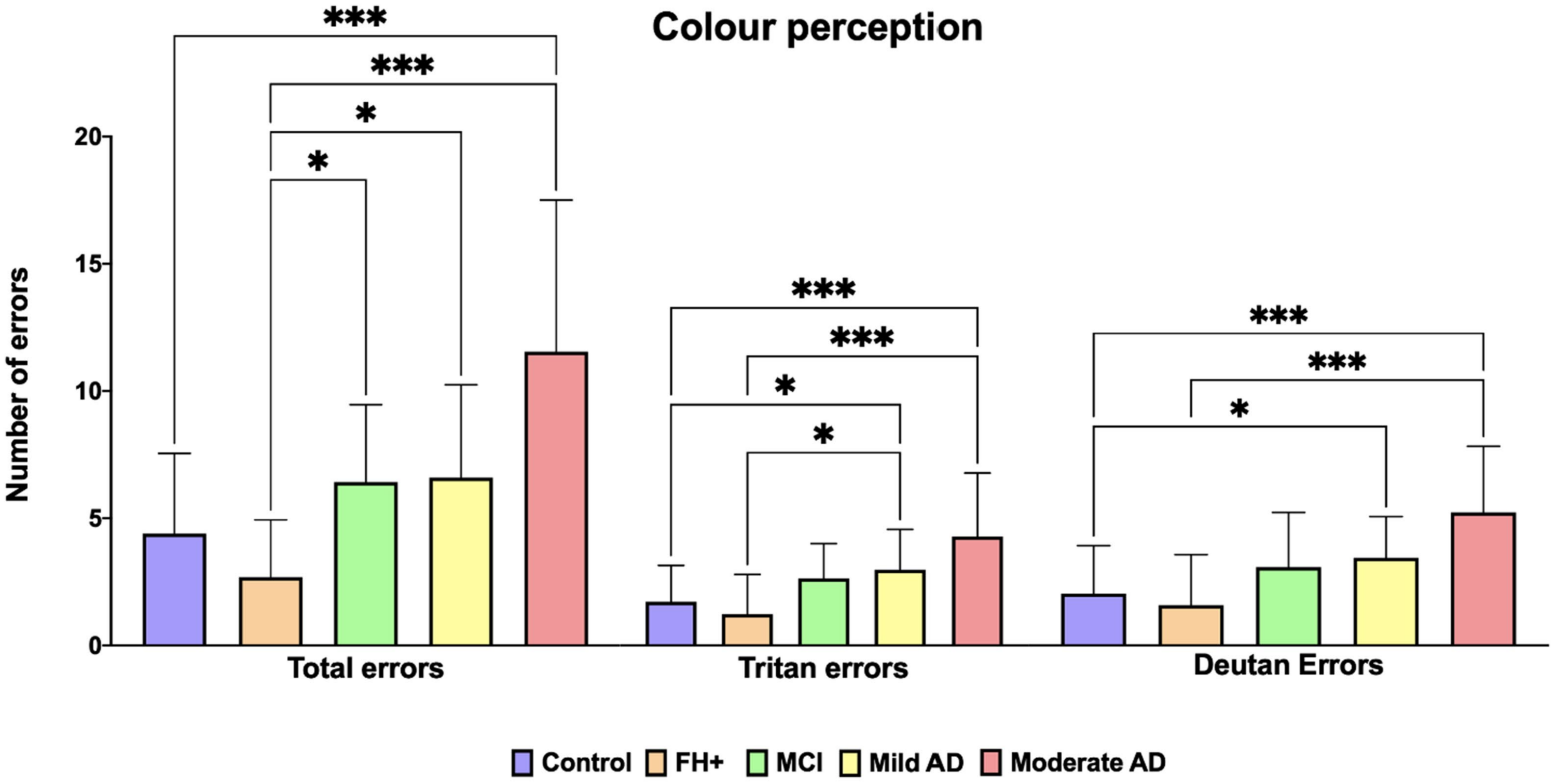
Median values of Farnsworth Roth 28-hue. FH+, family history positive; MCI, mild cognitive impairment; AD, Alzheimer’s disease. Each bar represents the media*n* ± interquartile range. *p < 0.05; ***p < 0.001.

In the Farnsworth 28-hue Test, the analysis of the number of total errors shown an increase in the number of errors between the control group and moderate AD group (value of *p*<0.001). We also found a significant increase in the number of total errors between FH+ group and: (i) MCI group (value of *p*<0.05); (ii) mild AD group (value of *p*<0.05) and; (iii) moderate AD group (value of *p*<0.001) (Figure 5).

When we analyze the tritan axis, we found significant differences between the control group and: (i) mild AD group (value of *p* <0,05) and; (ii) moderate AD group (value of *p*<0.001). We also found significant differences between FH+ group and: (i) mild AD group (value of *p*<0.05) and; (ii) moderate AD group (value of *p*<0,001) (Figure 5).

On the deutan axis, we found significant increase in the error number between control group and: (i) mild AD group (value of *p*<0.05) and; (ii) moderate AD group (value of *p*<0.0001). We also found statistical significance increase when comparing FH+ group to the moderate AD group (value of *p* <0.001) (Figure 5).

### 3.5. Perception digital test

**Figure 6:**
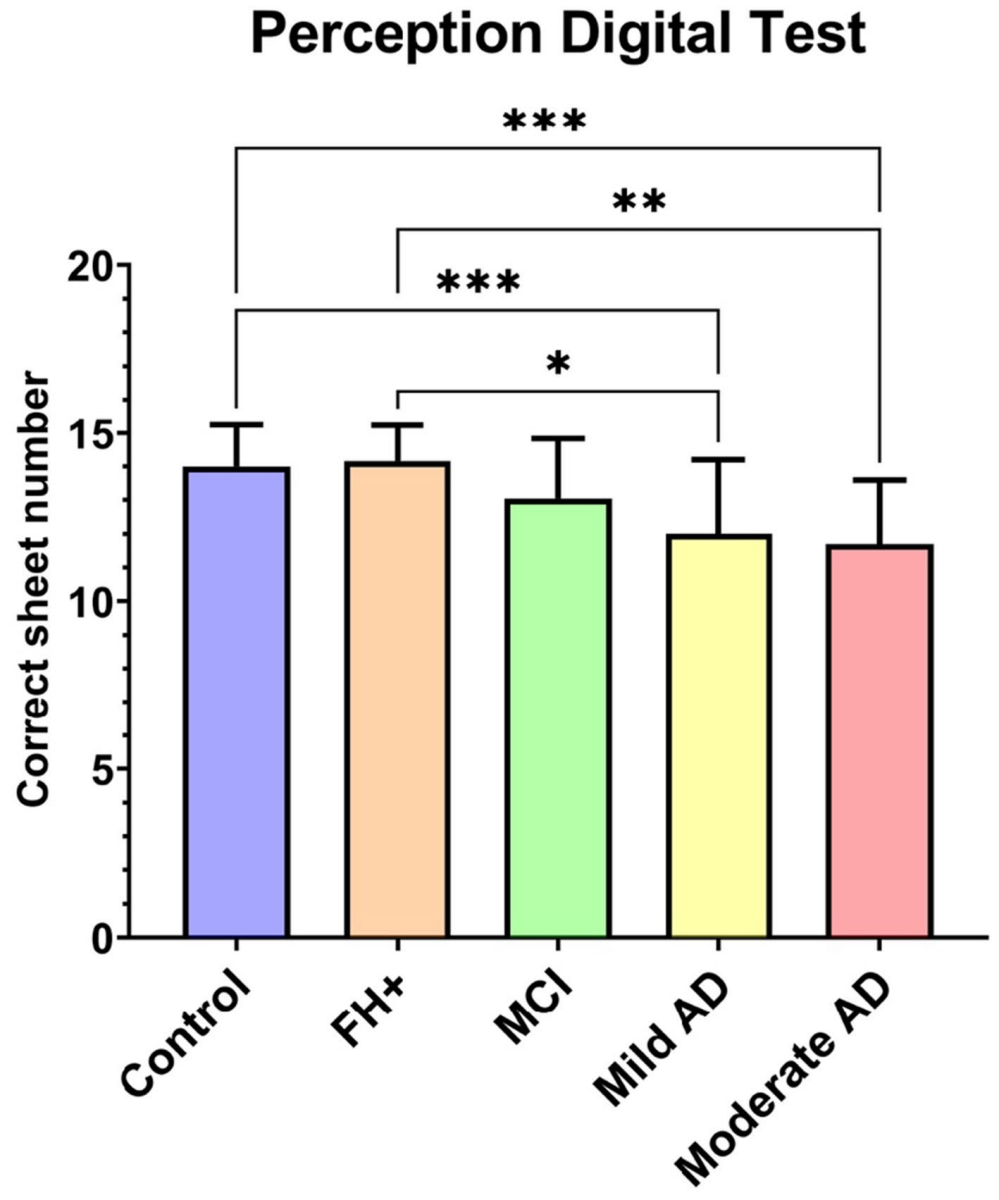
Median value of perception digital test between groups. FH+, family history positive; MCI, mild cognitive impairment; AD, Alzheimer’s disease. Each bar represents the media*n* ± interquartile range. *p < 0.05; **p < 0.01; ***p < 0.001.

In the Perception digital test (PDT) median values we found that in comparison with the control group there was a decrease in: (i) mild AD group and (ii) moderate AD group (*p* < 0.0001, in all instances). We also found statistical significance decrease when comparing FH+ group and: (i) mild AD (value of *p* <0.05) and; (ii) moderate AD (value of *p* <0.01) (Figure 6).

### 3.6. Correlation between MMSE and visual function

When analyzed the correlation between the MMSE score with the different visual function tests, we found a statistical significant direct correlation with: (i) VA (*r* = 0.402; value of *p*<0.001); (ii) CS in all spatial frequencies: 3 cpd (*r* = 0.4003; value of *p*<0.001); 6 cpd (*r* = 0.5634; *p* < 0.001), 12 cpd (*r* = 0.453; value of *p*<0.001) and; 18 cpd (*r* = 0.4182; value of *p* <0.001) and; (iii) PDT (*r* = 0.507; value of *p*<0.001).

Moreover, when we analyzed the correlation between the MMSE and the results in the Farnsworth color test, we found a statistically significant negative correlation in: total error number (*r* = −0.511; value of *p* <0.001); tritan axis errors (*r* = −0.448; value of *p* <0.001) and deutan axis errors (*r* = −0.461 and value of *p* <0.001).

### 3.7. Roc curves of the visual tests

**Figure 7:**
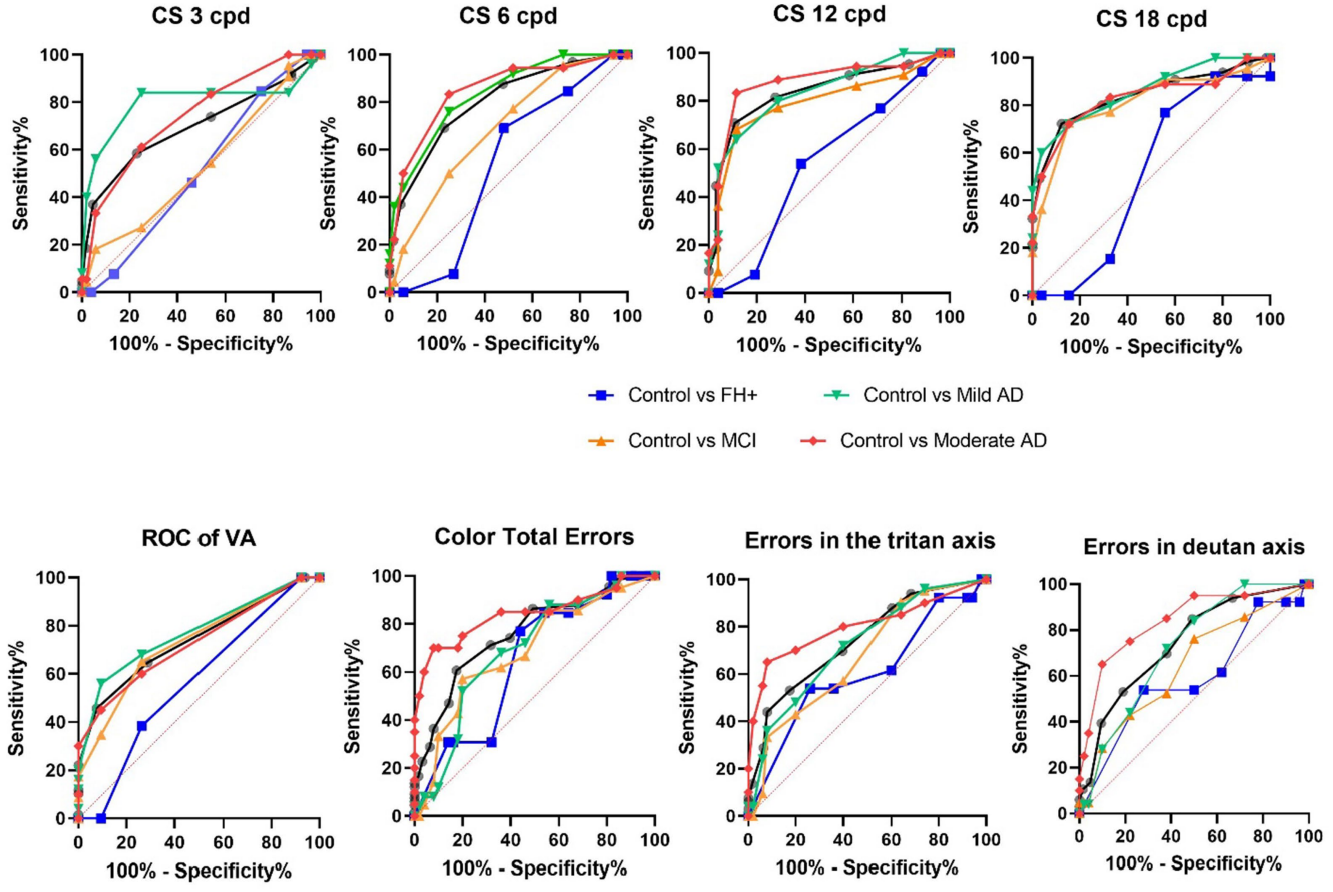
aROC curves of the different visual tests among the different study groups. FH+, family history positive; MCI, mild cognitive impairment; AD, Alzheimer’s disease.

The results indicated that the psychophysical tests had varying degrees of accuracy in discriminating between the different groups. For the cognitively healthy vs. cognitive decline comparison, all psychophysical tests showed statistically significant differences with *p*-values less than 0.01. The 18 cycles per degree (cpd) test had the highest aROC value at 0.8401, while the 3 cpd test had the lowest aROC value at 0.6991 (Table 2; Figure 7).

**Table 2:** aROC values of the different visual tests among the different study groups.

Area under the ROC curve		Cognitively healthy vs Cognitively decline	Control vs FH+	Control vs MCI	Control vs Mild AD	Control vs Moderate AD
VA	Area	0.7398	0.5653	0.7305	0.7713	0.7283
	*p* value	**<0.0001**	0.4681	**0.0015**	**0.0001**	**0.0028**
3 cpd	Area	0.6991	0.5155	0.5367	0.7988	0.7420
	*P* value	**<0.0001**	0.8633	0.6194	**<0.0001**	**0.0023**
6 cpd	Area	0.8002	0.5318	0.6888	0.8342	0.8478
	*P* value	**<0.0001**	0.7244	**0.0106**	**<0.0001**	**<0.0001**
12 cpd	Area	0.8378	0.5377	0.7911	0.8342	0.8750
	*P* value	**<0.0001**	0.6758	**<0.0001**	**<0.0001**	**<0.0001**
18 cpd	Area	0.8401	0.5118	0.8090	0.8600	0.8312
	*P* value	**<0.0001**	0.8956	**<0.0001**	**<0.0001**	**<0.0001**
Farnsworth total errors	Area	0.7618	0.6477	0.6886	0.6916	0.8410
	*P* value	**<0.0001**	0.1030	**0.0126**	**0.0071**	**<0.0001**
Farnsworth tritan errors	Area	0.7427	0.6038	0.6757	0.7216	0.8005
	*P* value	**<0.0001**	0.2516	**0.0201**	**0.0019**	**<0.0001**
Farnsworth deutan errors	Area	0.7439	0.5915	0.6462	0.7228	0.842
	*P* value	**0.0017**	0.3122	0.0531	**0.0017**	**<0.0001**

ROC, receiver operating characteristic; VA, visual acuity; cpd, cycles per degree; FH+, family history positive; MCI, mild cognitive impairment; AD, Alzheimer’s disease. In bold significant values.

The results pertaining to the predictive value of VA test indicate that individuals with cognitive decline and the MCI, Mild AD, and Moderate AD groups exhibit a predictive value greater than 0.7283 when compared to the control group (Table 2; Figure 7).

When comparing the control group to those with MCI, mild AD, and moderate AD, the 12 cpd and 18 cpd tests showed the highest accuracy in distinguishing between groups with aROC values most above 0.8342. The Farnsworth total errors test also showed high accuracy, particularly in distinguishing between the control group and those with moderate AD with an aROC value of 0.8410 (Table 2; Figure 7).

The results of the study examining the Farnsworth Tritan Errors and Farnsworth Deutan Errors using the Area under the ROC curve reveal significant differences in the discriminative power of the Farnsworth tritan errors and Farnsworth deutan errors across the different comparisons. In the Cognitively Healthy vs. Cognitively decline comparison, both parameters demonstrate high discriminative power (*p* < 0.0001). However, in the Control vs. FH+ comparison, there is limited discriminative power for both parameters (*p* > 0.05). In the Control vs. MCI comparison, the Farnsworth tritan errors and Farnsworth deutan errors show moderate discriminative power (*p* < 0.05), while in the Control vs. Mild AD and Control vs. Moderate AD comparisons, both parameters exhibit significant discriminative power (*p* < 0.001) (Table 2; Figure 7).

## 4. Discussion

To our knowledge, this is the first study to analyze the visual function of 5 groups of subjects, corresponding to the different stages of the AD continuum (“Control,” “FH+,” “MCI,” “mild AD” and, “moderate AD”). The study sample was carefully selected, and all participants met the inclusion criteria in terms of diagnosis of neurological disease or any ocular disease that could interfere with the results.

When analysing the MMSE of the study groups, we found statistically significant differences between the control group, the FH+ group and the MCI group, which had a higher MMSE score than the mild and moderate AD groups. The control, FH+ and MCI groups did not differ from each other. In the sample analysed in the present study, the mild AD group had an MMSE of 25.00 (21.00–26.50), a higher score than values reported in other studies (Cronin-Golomb et al., 1995, 2007; Lakshminarayanan et al., 1996), these values indicate that they are patients with a very early stage of the disease. The MMSE score of our patients is very high because our patients were newly diagnosed, and therefore, it is an early phase in each of the stages. All patients had a diagnosis given by a team of geriatricians and neuropsychologists after an extensive battery of tests, including magnetic resonance imaging. We specifically chose this population to reliably answer visual tests, as psychophysical visual tests require patient collaboration, and although the examiners had extensive experience with patients with cognitive impairment, minimal patient involvement was required.

Although there were differences among the different age groups, all patients were over 65 years old, including those in the FH+ group. This inclusion criterion was chosen because it is known that visual function declines around this age range, and the population over 65 years old can be considered as a homogeneous group (Ross et al., 1985; Elliott, 1987; Liutkevičienė et al., 2013; Pelletier et al., 2016). It is noteworthy that subjects in the FH+ group had a younger age range because they were those whose parents had suffered from dementia, and although they had predisposing genetic traits, cognitive decline had not yet developed. That is precisely why the age of this group was lower, and it is interesting to understand what happens at this stage in order to detect possible early differences in visual function.

Patients with AD suffer from various symptoms at different stages, one of which is language problems, including nominative deficits. For this reason, the selection of visual tests was carefully considered in this study, choosing those with limited naming demands and easily performable by these patients. For the measurement of VA, the letters are presented in isolation for identification, as it has been observed that patients perform better on the test and obtain better results in this way (Sadun et al., 1987). Similarly, the analysis of CS with the CSV-1000E test, which has a low influence of VA compared to other existing CS tests (Neargarder et al., 2003) and the colour perception with the Roth 28-hue test that did not require verbalisation of the results by the patients (Berry, 2017; Salobrar-García et al., 2019).

A previous study performed in youngest AD relatives (average age of 56 years old), it was showed a hypersynchronization in functional connectivity measured by magnetoencephalography of high alpha band in comparison with the control group (Ramírez-Toraño et al., 2021). This alteration is caused by a possible imbalance of excitation/inhibition of GABAergic neurons located in the vicinity of Aβ plaques that are deposited very early in AD (Garcia-Marin et al., 2009; Busche and Konnerth, 2016). In the present study, we analysed the visual function of subjects with a family history of AD and over 65 years of age, to test whether there are similar changes in the visual pathway as have been observed in the electric response of the brain.

The results obtained in VA showed a significant decrease in the MCI, mild and moderate AD patients with respect to the control group. This alteration that appears in both preclinical and early stages of the disease could be due to an alteration in the function of neurotransmitters, specifically acetylcholine. It is known that acetylcholine has a crucial role in the peripheral and central nervous systems, and in AD cholinergic neurons, are severely lost in AD (Ferreira-Vieira et al., 2016). In the retina, these neurotransmitter changes are mainly evident in the photoreceptor layer and in the inner nuclear layer and are associated with the loss of cholinergic cells in the retina (Nobili and Sannita, 1997; Schliebs and Arendt, 2006; Oliveira-Souza et al., 2017; Cerquera-Jaramillo et al., 2018). In the literature, according with our results, we found studies in which this loss of VA appears also in patients with cognitive decline and worsens as the disease progresses (Sadun et al., 1987; Salobrar-García et al., 2019; Rehan et al., 2021; Chang et al., 2022; Wu et al., 2022). Also, in the present work, we found a positive correlation between the MMSE score and the VA.

VA and CS impairment may be risk factor for cognitive decline (Ward et al., 2018; Swenor et al., 2019; Smith et al., 2021) and they may precede the onset of clinical cognitive impairment several years earlier. These findings suggest that reduced VA may be an early manifestation of central nervous system degeneration and/or that impaired visual function may contribute to cognitive impairment (Brenowitz et al., 2019; Naël et al., 2019; Swenor et al., 2019; Tran et al., 2020). Also the reduction in VA can be explained by cortical and subcortical dysfunction of the visual system further affecting writing, reading and face recognition (Cerquera-Jaramillo et al., 2018).

One of the earliest visual manifestations in patients with AD, are alterations in CS (Risacher et al., 2013; Jindal, 2015), which have been associated with impairment in more cognitive domains than other measures of visual functioning (Varadaraj et al., 2021). CS worsens throughout the course of the disease, and is related to damage to the magnocellular pathway of the lateral geniculate nucleus of the central nervous system (Sartucci et al., 2010; Risacher et al., 2013). Epidemiological studies have shown that older adults with impaired CS are at increased risk of cognitive impairment at 10 years of follow-up (Fischer et al., 2016). It was also reported a strong association between brain areas involved in the disease with CS records which may be predictive of abnormal Aβ and p-Tau protein accumulation. In addition, a reduction in CS, measured by frequency doubling technology, has been associated with Aβ and P-Tau brain deposition as well as neurodegeneration, both throughout disease progression and alone in subjects at high risk for AD development (Risacher et al., 2020). In our study there are significant decreases in CS at both, low and high frequencies, between: (i) the control group and the AD groups and, (ii) the FH+ group and the AD groups; as reported in other studies in the literature associated with the MMSE score (Risacher et al., 2013; Salobrar-García et al., 2019). This correlation also has been found in the present study where in addition, we observed that high frequencies are decreased in all groups except for the comparison between the control vs. FH+ group. This decrease therefore already appears in the MCI group. The greatest reduction at higher spatial frequencies has been described by several authors (Hutton et al., 1993; Gilmore and Whitehouse, 1996; Salobrar-García et al., 2015, 2019), but others report that the higher reduction occurs at low spatial frequencies (Levine et al., 1993; Baker et al., 1997; Cronin-Golomb et al., 2007; Polo et al., 2017) and others find no differences with respect to the control group (Schlotterer et al., 1984; Rizzo and Nawrot, 1998; Massoud et al., 2002). The discrepancies observed in the literature in the analysis of CS at different stages of the disease may be due to the heterogeneity of the samples and the tests used to analyze it (Neargarder et al., 2003).

Another of the manifestations in AD patients are alterations in colour perception. These differences could be mainly due to participation both the parvocellular pathway, which is characterized by small axons of the optic nerve (Salamone et al., 2009) and the koniocellular pathway which is involved with the blue-yellow spectrum (Martin et al., 1997). One of the alterations linked to AD is a notable decrease in the cortical region V4, which plays a crucial role in the processing of chromatic information (Chan et al., 2001; Brewer and Barton, 2016). On the other hand, changes in colour vision in AD have been associated with variations in different retinal layers according to Köllner’s rule. While changes in the retinal ganglion cells, optic nerve, visual pathway and visual cortex lead to a deficiency in the red-green axis, changes in the outer retina contribute to alterations in the blue-yellow axis (Huna-Baron et al., 2013; Kim et al., 2022). On the other hand, in the AD, it has been described that there is an degeneration on the photoreceptor cells that does not occur in only one type of cone, which is due to the reduction of melatonin levels and its antioxidant effects that occurs in AD (Savaskan et al., 2002).

When we analyzed colour perception in our patients, we observed that MCI, mild AD and moderate AD had a significant increase in the number of total errors in comparison to FH+ group. Also, the moderate AD group showed a significant increase in the total number errors related to control group. This diffuse involvement has been observed by different authors (Pache et al., 2003; Salamone et al., 2009; Polo et al., 2017; Salobrar-García et al., 2019) and recently Vidal et al. had found that individuals with AD and those with MCI display an acquired color vision deficiency, both in protan and tritan axism that is likely associated with compromised brain metabolism (Vidal et al., 2022).

In our patients, we found in the tritan axis errors, significant differences between the control group and mild and moderate AD groups, as well as between the FH+ and mild and moderate AD. However, we found no significant differences in this axis between the MCI group and the control group. In contrast, a loss in the tritan axis has been reported between the MCI subjects and the control group (Vidal et al., 2022). The MCI group is an intermediate stage between normal subjects and AD patients (Lewis et al., 1987; Black, 1996) that have worse MMSE scores (Vidal et al., 2022). In the present work, in all participants, we observed a negative correlation between the MMSE score and the total error number, tritan and deutan axis.

With respect to the deutan axis, we found statistically significant differences when we compared between the control group and both mild and moderate AD and when we compared between the FH+ and moderate AD groups. Similar than Vidal et al. (2022), we also found no significant differences in the number of errors between the control and MCI groups.

In a previous work, we found that in subjects with moderate AD the PDT showed a high direct correlation with the MMSE score and the aROC curves showed a good prognostic value (Salobrar-García et al., 2019). In the present study, our MCI patients showed no differences in PDT, however there were statistically significant differences between the mild and moderate AD groups and the control and FH+ groups. In addition, we also found a statistically significant positive correlation between the MMSE score and PDT. These results could be explained due to alterations in the visual processing in the AD pathology, that would take place in the regions involved in the magnocellular pathway (parietal and frontal brain areas) (Bar, 2003; Saumier et al., 2005).

Overall, our results of the aROC curves suggest that some psychophysical tests may be useful in identifying individuals with cognitive decline or disease, with the CS test analysing the 12 cpd and 18 cpd showing particular promise in distinguishing between control groups and those with mild or moderate AD.

As all the research works, this study shows strengths and some limitations. One of the first limitations is the low number of participants in each of the study groups. However, it should be noted that these have been carefully selected and that all participants met strict ophthalmological and memory criteria. We could be sure that all visual defects found are due to neurodegeneration and not to a visual problem. Despite having no biological biomarkers of the disease, the clinical diagnosis of the participants was made by professionals who followed standard criteria. It would be interesting for future studies to have bigger samples and to be able to correlate them with functional findings. On the other hand, it would be very interesting to carry out longitudinal studies of participants at high genetic risk for the development of AD and patients with MCI, so that we could learn about the evolution of the functional changes found in these early stages of the pathology. One of the strengths of this study is that the tests used in the present study to analyse visual function are easy to apply and could be useful together with neuropsychological tests and imaging tests for the diagnosis of AD, however, we are aware that other tests could be used for the analysis of visual function such as pupillometry, analysis of extraocular movements, and other tests for the analysis of visual function.

In conclusion, alterations in visual function appear already in subjects with MCI and evolve when AD disease is established. These differences seem to remain stable between the different early stages of AD (mild and moderate AD). Although in the present study we found no differences in visual function in the FH+ group, it would nevertheless be interesting to carry out longitudinal studies in these population to find out whether they develop the disease in the future. Therefore, visual psychophysical tests are a useful, simple and complementary tool to neuropsychological tests to facilitate diagnosis in the preclinical and early stages of AD and they correlated with the cognitive function.

## Data availability statement

The raw data supporting the conclusions of this article will be made available by the authors, without undue reservation.

## Ethics statement

The studies involving human participants were reviewed and approved by the research followed the tenets of the Declaration of Helsinki, and the studies were approved by the local ethics committee (HCSC) with the internal code 11/372-E, 18/422-E_BS and 20/698-E_Tesis. The patients/participants provided their written informed consent to participate in this study.

## Author contributions

LE-H, IL-C, RH, MS, MD-L, AR, JS, FM, PG, JR, and ES-G: conceptualization and validation. LE-H, IL-C, RH, MS, LS-P, FR-T, JM, JF-A, PR, SA, MD-L, AR, JS, FM, PG, JR, and ES-G: methodology. LE-H, IL-C, RH, MS, FR-T, SA, MD-L, AR, JS, FM, PG, JR, and ES-G: formal analysis. LE-H, IL-C, RH, MS LS-P, FR-T, JM, JF-A, PR, SA, MD-L, AR, JS, FM, PG, JR, and ES-G: investigation. RH, MD-L, AR, JS, FM, PG, and JR: resources. LE-H, IL-C, MS, LS-P, FR-T, JM, JF-A, PR, SA, and ES-G: data curation. LE-H, IL-C, RH, LS-P, MD-L, AR, JR, and ES-G: writing—original draft preparation, LE-H, IL-C, RH, AR, JS, JR, and ES-G: writing—review and editing. RH, JR, and ES-G: supervision. RH, MD-L, AR, JS, FM, PG, JR, and ES-G: project administration. RH, FR-T, AR, JS, FM, PG, and JR: funding acquisition. All authors contributed to the article and approved the submitted version.

## Funding

This research was funded by the Research Network RETIBRAIN (RED2018-102499-T) of the Spanish Ministry of Science and Innovation; and the Spanish Ministry of Economy and Compet-itiveness (Grant PSI2015-68793-C3-1-R) and by the Spanish Ministry of Economy and Competi-tiveness under the Grant PSI2015-68793-C3-1-R [D601], by the project B2017/BMD-3760 from NEUROCENTRO. IL-C was currently supported by a Postdoctoral Fellowship (CT42/18-CT43/18) from the Complutense University of Madrid. JF-A was currently supported by a Predoctoral Fellowship (FPU17/01023) from the Spanish Ministry of Science, Innovation, and Universities. LS-P was currently supported by a Predoctoral Fellowship (CT82/20-CT83/20) and JM was currently supported by a Predoctoral Fellowship (CT58/21-CT59/21) from the Complutense University of Madrid.

## Conflict of interest

The authors declare that the research was conducted in the absence of any commercial or financial relationships that could be construed as a potential conflict of interest.

## Publisher’s note

All claims expressed in this article are solely those of the authors and do not necessarily represent those of their affiliated organizations, or those of the publisher, the editors and the reviewers. Any product that may be evaluated in this article, or claim that may be made by its manufacturer, is not guaranteed or endorsed by the publisher.
